# Azimuth mapping of fibrous tissue in linear dichroism-sensitive photoacoustic microscopy

**DOI:** 10.1016/j.pacs.2023.100510

**Published:** 2023-05-10

**Authors:** Eunwoo Park, Yong-Jae Lee, Chulhong Kim, Tae Joong Eom

**Affiliations:** a Department of Convergence IT Engineering, Electrical Engineering, Mechanical Engineering, Medical Science and Engineering, Graduate School of Artificial Intelligence, and Medical Device Innovation Center, Pohang University of Science and Technology (POSTECH), Pohang, Gyeongbuk 37673, the Republic of Korea; b Engineering Research Center for Color-Modulated Extra-Sensory Perception Technology, Pusan National University, Busan 46241, the Republic of Korea; c Department of Congo-Mechatronics Engineering, Pusan National University, Busan 46241, Republic of Korea

**Keywords:** Dichroism, Photoacoustic microscopy, Polarization, Anisotropy, Optic axis, Stokes parameters

## Abstract

Photoacoustic imaging (PAI) has emerged as a molecular-selective imaging technology based on optical absorption contrast. Dichroism-sensitive photoacoustic (DS-PA) imaging has been reported, where the absorption coefficient has a vector characteristic, featuring dimensions of contrast in polarization and wavelength. Herein, we present a DS-PA microscopy (DS-PAM) system that implements optical anisotropy contrast and molecular selectivity. Moreover, we propose mathematical solutions to fully derive dichroic properties. A wavelength for the PAI of collagenous tissue was used, and the proposed algorithms were validated using linear dichroic materials. We successfully mapped dichroic information in fibrous tissue imaging based on the degree of anisotropy and axis orientation, and also deduced mechanical assessment from the tissue arrangement. The proposed DS-PAM system and algorithms have great potential in various diagnostic fields using polarimetry, such as musculoskeletal and cardiovascular systems.

## 1 Introduction

Polarization is a contrast-enhancing indicator for optical sensing. Since several materials exhibit polarization specificity, such as that associated with birefringence, various optical imaging modalities have been used to evaluate the anisotropic characteristics qualitatively and quantitatively [1], [2], [3], [4], [5]. Polarization-sensitive optical coherence tomography (PS-OCT), polarized light microscopy (PM), and second-harmonic generation microscopy have been prominently used for tissue polarimetry and visualization of fibrous tissues in biomedical applications [6], [7], [8], [9], [10], [11], [12], [13], [14]. These polarization contrast imaging modalities can be used to observe diagnostic biomarkers, including molecular morphological and biomechanical features.

Photoacoustic (PA) imaging (PAI) has become a widely used three-dimensional (3D) imaging technology with high biomolecular selectivity. The PA effect relies on the optical absorption properties of the chromophore, and the absorption contrast enables structural and functional biomedical imaging from the molecular to organ level [15], [16], [17]. However, in conventional PAI, the optical absorption coefficient is regarded as a function of wavelength and treated as a scalar variable [18], [19], [20], [21]. The wavelength-prospective (spectral) approach in PAI makes it possible to distinguish the composition of biological tissues, such as nuclei, hemoglobin, collagen, and lipids, and their concentrations [22], [23], [24], [25], [26], [27], [28]. Thus, most selective PAI-based pathological diagnoses are performed by morphological and hemodynamic analyses of the surrounding tissue rather than the intuitive analysis of the lesion itself [29], [30], [31], [32], [33].

Recently, a dichroism-sensitive (DS-) PAI that can add polarization contrast to PA images has been reported [34], [35], revealing its potential in tissue polarimetry. The term dichroism refers to the anisotropic selective optical absorption property for different polarization states of the incident light. In dichroic materials, the optic axis, or principal axis, is determined by the arrangement of atoms in them, and the electric field components of the incident wave perpendicular thereto are strongly absorbed in the medium [36]. The anisotropy of the binding forces due to the asymmetric conformation appears as anisotropy of the natural frequencies of the atoms. When linearly polarized light interacts with dichroic molecules in biological tissues, the difference between the natural frequency and the electric field frequency determines the speed of the propagating wave and thus the complex refractive index. Consequently, according to the Kramers–Kronig relations, the birefringent characteristics change the optical absorption bands depending on the polarization state of the incident light, and this phenomenon is directly reflected in the PA signal. In particular, based on the linear dichroism-based PA effects, DS-PAI enables the visualization of optical absorption anisotropy in fibrous tissues.

Unlike computed tomography systems (DS-PACT) [35], general PA microscopy (PAM) systems use a single-element ultrasonic transducer (UST), constraining repetitive acquisition and time [37]. Therefore, DS-PAM systems should perform a polarization analysis with an optimized number of acquisition angles. Previous studies on DS-PAM have suggested visualizing dichroism-related parameters, including the degree of anisotropy and axis orientation [34], [38], [39]. However, an insufficient number of acquisition angles does not allow the proper calculation of the parameters, resulting in misinterpretation of dichroism and azimuth. The azimuth, which implies the orientation of molecular alignment, is necessary to comprehend tissue polarimetry and macroscopic molecular orientation changes in fibrous tissue, such as tendons and ligaments, which are commonly observed in living organisms [40], [41], [42]. To fully map DS-PA parameters, adequate computational methods need to be devised.

In addition, previous studies used visible wavelength light sources, which were ineffective for molecular selectivity in DS-PAI. For the wavelengths in the visible band, the light absorbance is substantially low in typical chromophores having fibrous structures. Thus, the visible band DS-PAI relies solely on absorption changes from polarization states, hindering sensitivity. As an example of a fibrous structure, collagen has been reported to have PA absorption peaks in a short-wave infrared (SWIR) band (e.g., ∼1200, ∼1500, and ∼1700 nm), which may improve the sensitivity of DS-PAI [25], [43].

In this paper, we present DS-PAM that combines spectral contrast and polarization contrast. Moreover, we propose methods for dichroic parameter mapping with appropriate acquisition angles. The SWIR band light source enables collagenous tissue PAI, and mapping algorithms are used to visualize the DS-PA parameters. The proposed system can provide a biomechanical interpretation of fibrous arrangements. The selective DS-PAM system and azimuth mapping algorithms can make breakthroughs in tissue polarimetry through dual-contrast implementation.

## 2 Materials and methods

### 2.1 Experimental setup

**Fig. 1:**
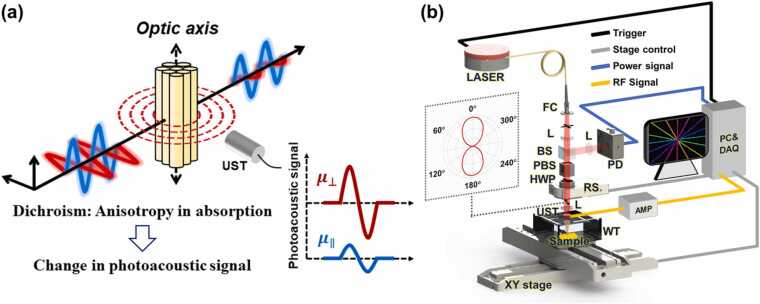
Dichroism-sensitive photoacoustic microscopy (DS-PAM). (a) The principle diagram of the linear dichroism-based photoacoustic effect. (b) The SWIR-DS-PAM system configuration. FC, fiber collimator; L, lens; BS, beam splitter; PBS, polarizing beam splitter; RS, rotary stage; UST, ultrasonic transducer; WT, water tank; AMP, amplifier; PD, photodetector; PC, personal computer; DAQ, data acquisition.

To demonstrate the linear dichroic PA effect (Fig. 1a), a DS-PAM system was constructed, as depicted in Fig. 1b. A nanosecond pulsed fiber laser (CoLID-I, Connet, China) with a central wavelength of 1540 nm, a pulse repetition rate of 1 kHz, and pulse width of approximately 20 ns was used as the light source. The power stability of the laser output was maintained using an active air-cooling breadboard (PTC1/M, Thorlabs, USA) and a thermal pad. This laser wavelength is suitable for the PAI of collagen-based fibrous tissues [25]. A polarizer (GL15-C, Thorlabs, USA) was used to ensure linear polarization of the output laser beam, and the polarized light was modulated through a half-wave plate (HWP, WPH10ME-1550, Thorlabs, USA) on a rotating mount (K10CR1, Thorlabs, USA). The inset polar coordinate graph in Fig. 1b represents the angle dependence of the laser output intensity, proving polarization linearity. Linearly polarized light was focused and irradiated onto the sample passing through the hole of a ring-shaped ultrasonic transducer (UST, custom-made, Precision Acoustics, UK). A UST with a center frequency of about 20 MHz and an acoustic focal length of 10 mm was used to detect the PA signal, and the acquired signals were amplified by a 56-dB gain using two amplifiers (ZFL-500LN, Mini-Circuits, USA) in series. The amplified analog PA signal was converted to a 14-bit digital signal using a digitizer (PX14400, Signatec, USA) with a sampling rate of 400 MHz. A photodetector (PDA05CF2, Thorlabs, USA) simultaneously captured the pulse energy to compensate for laser power fluctuations. An XY motorized stage (DDS220/M, Thorlabs, USA) was used to convey the sample.

### 2.2 Principle of dichroism-sensitive photoacoustic imaging

The principle of DS-PAI is described below. Considering a vectorial optical absorption, the PA local pressure p is expressed as follows [35], [39]:

(1) $$\begin{array}{c} p\left(\theta ,\phi \right)=\Gamma \eta_{th}\mu_{a}\left(\theta ,\phi \right)F\left(\phi \right)\\ =\Gamma \eta_{th}F\cdot \left[\frac{\mu_{∥}+\mu_{⊥}}{2}+\frac{\mu_{∥}-\mu_{⊥}}{2}\;\cos\left(2\phi -2\theta \right)\right] \end{array},$$

where Γ is the Grüneisen parameter, $\eta_{th}$ is the constant of the energy conversion efficiency from photon to phonon, and F(ϕ) is the laser fluence illuminated on a sample. The laser fluence can be treated as a constant if its value is maintained constant regardless of the polarization angle. $\mu_{a}\left(\theta ,\phi \right)$ is the vectorial optical absorption coefficient that is represented by a combination of $\mu_{∥}$ and $\mu_{⊥}$. Moreover, $\mu_{∥}$ and $\mu_{⊥}$ are the optical absorption coefficients of the parallel and perpendicular polarized lights with respect to the orientation of the optic axis *θ*, respectively. ϕ is the linear polarization angle of the incident light, which is twice the mechanical rotation angle of the HWP.

#### 2.2.1 Trigonometric substitution method

The DS-PA signal was obtained by rotating the linear polarization angle of the incident light, and the azimuth of the sample was directly calculated using trigonometric identities. Previous studies on DS-PAM used two or three polarization angles to calculate the dichroism and azimuthal angle [34], [38]; however, they did not obtain the exact azimuth. The sine and cosine functions have periodicity and symmetry within a single period. The azimuth must be calculated by considering the sign between the two sinusoidal functions. Because the polarization state is symmetric with respect to a straight line (180°), the relationship between the supplementary and complementary angles can be expressed as follows:

(2) $$\begin{array}{c} p\left(\theta ,\;\phi_{a}\right)-p\left(\theta ,\;\pi -\phi_{a}\right)=\Gamma \eta_{th}F\cdot \left(\mu_{∥}-\mu_{⊥}\right)\cdot \;\sin\;2\phi_{a}\;\sin\;2\theta \;\left(\phi_{a}\neq m\pi ,\;m:\mathrm{integer}\right)\\ p\left(\theta ,\phi_{b}\right)-p\left(\theta ,\frac{\pi }{2}-\phi_{b}\right)=\Gamma \eta_{th}F\cdot \left(\mu_{∥}-\mu_{⊥}\right)\cdot \;\cos\;2\phi_{b}\;\cos\;2\theta \;\left(\phi_{b}\neq \frac{n\pi }{4},\;n:\mathrm{oddinteger}\right) \end{array}.$$

Several conditions are required to avoid singularities in the subsequent calculation process. Then, we can obtain the azimuth using the following equation:

(3) $$\theta =\frac{1}{2}\tan^{-1}\left(\frac{\frac{1}{N_{a}}\sum_{i}^{N_{a}}\frac{p\left(\theta ,\;\phi_{a}\left(i\right)\right)-p\left(\theta ,\;\pi -\phi_{a}\left(i\right)\right)}{\sin\;2\phi_{a}\left(i\right)}}{\frac{1}{N_{b}}\sum_{j}^{N_{b}}\frac{p\left(\theta ,\;\phi_{b}\left(j\right)\right)-p\left(\theta ,\;\frac{\pi }{2}-\phi_{b}\left(j\right)\right)}{\cos\;2\phi_{b}\left(j\right)}}\right),$$

where *N* is the number of angle sets corresponding to the subscripts, and *i* and *j* are the element indices of the acquired angle set. When $\phi_{a}=\phi_{b}$, the minimum number of linearly polarized light angles to solve the azimuth is three, for example, 30°, 60°, and 150°. However, this method is not suitable for direct derivation as the dichroism value has to be calculated by substituting the azimuth values backward. In addition, PA signals from biological tissues are typically weak and easily affected by ambient signals or disturbances. Because the azimuth values in this passive stepwise calculation are prone to misrepresent dichroism values, an alternative active mapping method is required.

#### 2.2.2 Stokes parameters in DS-PAM

When expressing Eq. (2) with four angles, that is, 0°, 45°, 90°, and 135° (or −45°), these angles can also determine the Stokes parameters I, Q, and U in terms of DS-PAI as follows [39]:

(4) $$S_{PA}=\left(I_{PA}Q_{PA}U_{PA}V_{PA}\right)=\left(p_{H}+p_{V}p_{H}-p_{V}p_{P}-p_{M}p_{R}-p_{L}\right)$$

where *p* is the amplitude of the PA signal with polarization states represented by various subscripts (*H*: horizontal linear (0°), *V*: vertical linear (90°), *P*: + 45° diagonal linear, *M*: − 45° diagonal linear, *R*: right circular, and *L*: left circular). According to the traditional Strokes parameter, the degree of linear polarization (DoLP) is defined as $\sqrt{Q^{2}+U^{2}}/I$. By developing an expression for the DoLP with PA Stokes parameters based on Eq. (4), the DoLP in DS-PAI reaches the value corresponding to linear dichroism:

(5) $$\mathrm{Dichroism}:\;\;\left|\frac{\mu_{p}-\mu_{v}}{\mu_{p}+\mu_{v}}\right|=\frac{\sqrt{Q_{PA}^{2}+U_{PA}^{2}}}{I_{PA}}.$$

In addition, the angle of linear polarization (AoLP) can also derive the azimuth in DS-PAI:

(6) $$\mathrm{Azimuth}:\;\theta =\frac{1}{2}\tan^{-1}\left(\frac{U_{PA}}{Q_{PA}}\right).$$

## 3 Results

### 3.1 Principle validation

**Fig. 2:**
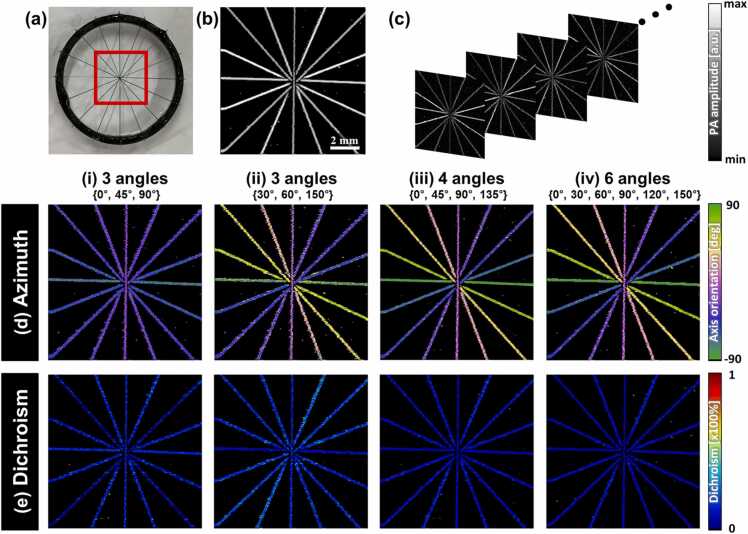
Validation of DS-PAM image reconstruction using the trigonometric method. (a) Photograph of crossed wires. The red box indicates the FOV for DS-PAM. (b) Conventional PA MAP image. (c) Series of images according to the polarization states. (d) Azimuth and (e) dichroism mapping according to (i–iv) the number of angles applied.

We demonstrated the proposed mapping algorithms after verifying the imaging system performance of the SWIR-DS-PAM (Supplementary Fig. S1 and Fig. S2). To mimic the fibrous structure with dichroism, 165-μm-thick dark-colored nylon monofilament wires were crossed, as shown in Fig. 2a. In addition, Fig. 2b shows a conventional PA maximum amplitude projection (MAP) image with unpolarized light, indicating the morphology of the sample. The field of view (FOV) was set to 10 × 10 mm^2^. By rotating the linearly polarized light (Fig. 2c), the axis orientations of the wire were calculated using the azimuths. Fig. 2d shows the azimuth map according to the number of components in a set of acquisition angles obtained by applying the trigonometric substitution mapping method of Eq. (3). Subsequently, dichroism was mapped, as shown in Fig. 2e. When two angles are considered, 0° and 90°, the azimuth cannot be expressed, and the dichroism value can be considered the absolute value of the difference between the two images. When using three linear polarization angles of 0°, 45°, and 90° (Fig. 2ⅰ), proper mapping is limited, presenting only half of the axis range of π/2 owing to the singularity in Eq. (2), that is, the symmetry of the trigonometric functions. In contrast, using angle sets of 30°, 60°, and 150° (Fig. 2ⅱ), we successfully mapped the entire azimuth axis in the range of π. Furthermore, using four and six angles (Fig. 2ⅲ–ⅳ), the azimuth and dichroism maps were obtained with high uniformity (Supplementary Fig. S3).

**Fig. 3:**
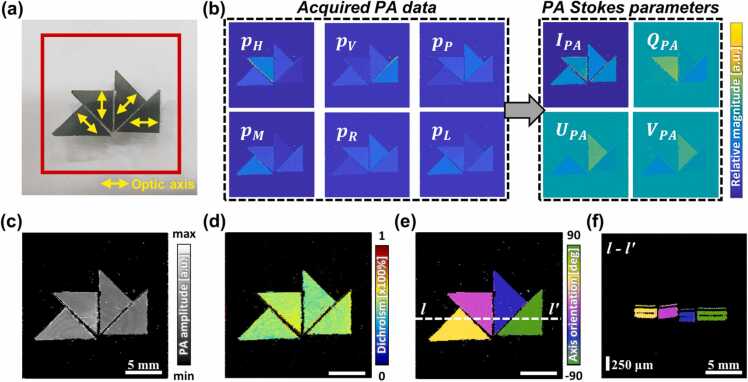
3D DS-PAM image reconstruction using Stokes parameters. (a) Photograph of polarizing films. Yellow arrows indicate the polarization axis of each film. (b) Stokes parameters in PAI. The color bar gives the magnitude of the signal: normalized PA amplitude (minimum to maximum) for the acquired data image, normalized magnitude (0−1) for I, and normalized magnitude (−1 to 1) for *Q*, *U*, and *V*. (c) Averaged PA MAP. (d) The dichroism image. (e) The PA azimuth map is represented by the optic axis of the polarizing film in MAP. (f) Cross-sectional PA azimuth map of the plane marked by the white dashed line *l–l′* in (e).

To obtain the azimuth and dichroism separately, we applied the Stokes parametric mapping method. A near-infrared linear polarizing polymer film (12–472, Edmund Optics, USA) was used, as shown in Fig. 3a. The yellow arrows in the inset indicate the polarization axis of each element, while the red box indicates the FOV set to 20 × 20 mm^2^. Fig. 3b shows PA MAP images for the polarization state of the incident light. In particular, changes in the PA signal amplitude according to the correlation with the optical axis of each element were identified. Perpendicular axes demonstrated stronger PA signals, whereas parallel axes appeared weak. The acquired PA data comprised the PA Stokes parameters. A quarter-wave plate (WPQ10ME-1550, Thorlabs, USA) was temporarily used to create the circularly polarized components. Furthermore, DS-PA analyses were performed using the linear PA Stokes parameters I, Q, and U. Fig. 3c shows the average PA MAP image according to the acquisition angle, representing the conventional PA structural MAP image. Fig. 3d shows the degree of anisotropy calculated from Eq. (5), as the linear dichroism value of the sample. All the film elements exhibited a dichroism value of approximately 0.55. Fig. 3e and f depict the orientation angles calculated using Eq. (6) as the azimuth in the top-view projection and cross-section, respectively. Azimuths were calculated using volumetric PA datasets, and the optic axis was correctly mapped.

### 3.2 Azimuth mapping of tendinous tissue

**Fig. 4:**
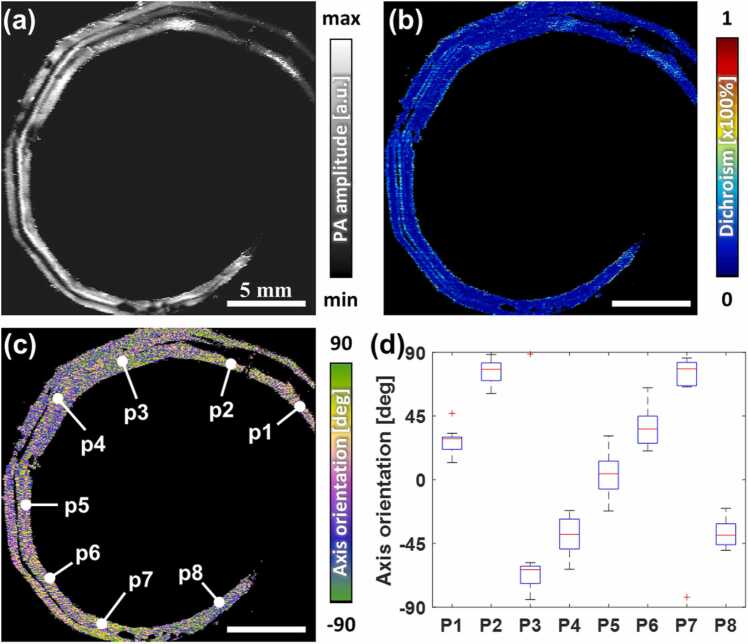
DS-PAM images of rat tail tendons. (a) Averaged PA MAP. (b) Dichroism map. (c) Azimuth map. (d) Boxplot of tendon azimuth distributions of white points in (c).

We applied the proposed system and method to rat tendons as biological samples. Animal experimental procedures were approved by the guidelines of the Institutional Animal Care and Use Committee at Chonnam National University Hospital (approval No. CNUHIACUC-20018). Male Sprague-Dawley rats (7 weeks old, weighing approximately 270 g) were sacrificed through carbon dioxide asphyxiation and cervical dislocation, and the tendons were collected. For a preliminary biological experiment, azimuth mapping was conducted using a rat tail tendon. The rat tail tendon was subdivided into 4–6 bundles located between the dermal layer and the caudal vertebra [44]. Fig. 4a shows the averaged PA MAP image of a rat tail. The curled tail was visualized with two connected tendon bundles because it was positioned with the dorsal side up. We reconstructed DS-PAM images using Stokes parameters. Fig. 4b and c show the dichroism and azimuth maps, respectively. The azimuth map shows the tail tendon bending. Detailed data were analyzed by setting regions of interest (ROIs) P1–P8 along a tendon bundle, as indicated in Fig. 4c. Fig. 4d shows the azimuthal distributions of tendons, and the axis orientations were identified according to the posture of the tail. The outliers at P3 and P7 appeared due to the π-period folding.

**Fig. 5:**
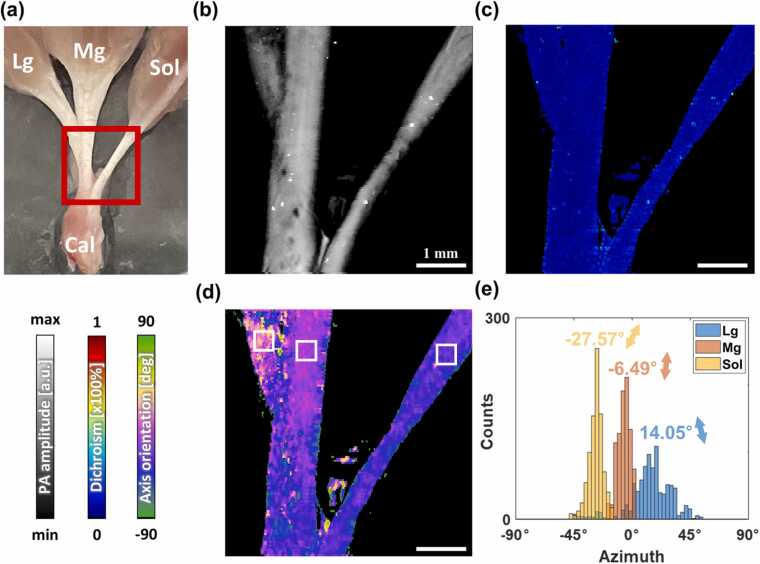
DS-PAM images of rat Achilles tendon. (a) Photograph. (b) Averaged PA MAP. (c) Dichroism map. (d) Azimuth map. (e) Histogram of tendon azimuth distribution in white boxes in (d).

The Achilles tendon consists of three sub-tendons connecting the calcaneus to the soleus (Sol), medial gastrocnemius (Mg), and lateral gastrocnemius (Lg) muscles [45]. As shown in Fig. 5a, each tendon is extended. The inset red box indicates the FOV set to 5 × 5 mm^2^. Fig. 5b–d show images of the averaged PA MAP, dichroism map, and azimuth map, respectively. The azimuth was mapped based on the orientation of the tendons. We analyzed the detailed orientation by setting ROIs on different tendons. The size of ROI was set to the same to secure comparability, and set to about 400 × 400 µm^2^ to sufficiently cover even the thin single-origin tendon. Fig. 5e shows the histogram of the azimuthal distribution for each ROIs marked with white boxes in Fig. 5d. The inset numbers indicate the mean value of the axis angle within the tendons, and the variance implies diversity. The distribution of the Lg tendon appeared to be wider than those of the others because the tendons of Sol and Mg were placed tightly, and that of Lg was placed loosely. The orientation mapping and mechanical characterization were successful.

## 4 Discussion and conclusion

In this study, we developed a SWIR-DS-PAM system that achieved spectral and polarization contrasts for fibrous structure imaging of tendons. We exploited a central wavelength of 1540 nm in SWIR to enable selective imaging of the collagenous tissue and adopted the linear dichroism mechanism to investigate optical anisotropy.

The light loss was substantially high when using water (H_2_O) as a matching medium due to the wavelengths of the SWIR band. Therefore, silicone oil with a kinematic viscosity of 1000 mm^2^/s and density of 0.97 kg/m^3^ was used as an acoustic propagation medium. The optical absorption coefficients of water and silicone oil at a wavelength of 1540 nm were measured to be approximately 11.9 cm^−1^ and 0.023 cm^−1^, respectively. The acoustic attenuation of silicone oil was measured to be 5.6 times higher than that of water when using the probe designed in this study. However, the probe’s efficiency for PA signal acquisition using silicone oil in SWIR was two orders of magnitude higher than using water.

Mathematical methods were proposed for mapping DS-PA images. By applying the trigonometric substitution mapping method, the degrees of anisotropy and azimuth of the full π range were obtained with sufficient acquisition angles. Furthermore, we revealed that DS-PAI can employ the Stokes polarization parametric approach. The Stokes parameters described the polarization state of light. By employing linear Stokes parameters, DoLP and AoLP derived the dichroism and azimuth, respectively. Both DS-PA images were accurately mapped compared to the PM images (Supplementary Fig. S4). In addition, the mechanical properties could be inferred via DS-PAI analysis. The macromolecular orientation of each tendon could be quantified, and its distribution from the polarization dispersion qualitatively determined the mechanical tension.

However, the proposed DS-PAI has some limitations. First, the laser output stability should be managed for reliable mapping of the DS-PA parameters. Because biological tissues have a low level of dichroism, they are prone to misinterpretation due to fluctuating laser fluence. Here, we managed the laser output using a thermal controller and photodetector, and the PA signals were calibrated using the laser fluence for each pixel according to the rotating incident light. Alternatively, single-shot imaging can provide benefits regarding acquisition time and reliability [38]. Second, polarization dispersion hinders the accuracy of DS-PA computations. Owing to the strong scattering or dispersion, the incident light cannot maintain its polarization state and depolarizes, passing through the biological tissue. On a positive note, scattering contrast-based optical imaging modalities have low penetration depths owing to a round-trip optical path, whereas DS-PAI can image deeper anisotropies beyond the ballistic regime [35]. For orientation axis mapping, multilayer structures with dichroic materials exhibit multi-order harmonic modulation frequencies [35], [46]. The phase determination of high-order modulation frequencies requires more acquisition angles and complex axis computations. Furthermore, deep learning-based undersampled PA reconstruction approaches can assist in addressing the challenges induced by increased acquisition and processing [47], [48], [49], [50].

DS-PAI can be integrated with PS-OCT for multidimensional analysis of optical anisotropy. PS-OCT and DS-PAI rely on the interaction of electric fields and molecular structural bonds, which affect the velocity and extinction terms in the complex refractive index expression. PS-OCT provides polarization contrast in OCT structural imaging of the scattering contrast. It represents tissue anisotropy with images of the degree of birefringence, phase retardation, axis orientation, and even diattenuation. Similarly, DS-PAI can provide tissue polarimetry for the high selectivity of PAI. Through multi-contrasts, the fusion of these two imaging modalities may provide profound insights into the complex refractive index changes in anisotropic tissues.

In conclusion, we proposed SWIR-DS-PAM combining birefringence properties and molecular selectivity for PAI without exogenous contrast agents. SWIR-DS-PAM, which identifies anisotropic molecular structures based on dichroism and azimuth, has great potential in various diagnostic fields such as dermatologic, musculoskeletal, cardiovascular, and nervous systems. In particular, SWIR-DS-PAM can be used as an intuitive diagnostic indicator for monitoring collagenous tissue healing or assessing fibrous connective tissue vulnerability.

## CRediT authorship contribution statement

**E.P**.: Conceptualization, Formal analysis, Methodology, Validation, Visualization. **Y.-J.L.**: Data curation and analysis, Methodology, Validation. **C.K.**: Funding acquisition, Resources, Supervision. **T.J.E.**: Funding acquisition, Project administration, Resources, Supervision. All authors discussed the results and contributed to the writing.

## Declaration of Competing Interest

The authors declare the following financial interests/personal relationships which may be considered as potential competing interests: C.K. has financial interests in Opticho, which, however, did not support this work. C.K. is a section editor of the journal Photoacoustics, but was not involved in the peer-review process of the manuscript. All other authors declare no conflict of interest.

## Data Availability

Data will be made available on request.
