# Supplementary material for: Azimuth mapping of fibrous tissue in linear dichroism-sensitive photoacoustic microscopy
Source: Photoacoustics. 2023 May 10;31:100510. doi: 10.1016/j.pacs.2023.100510 (PMC10203768; doi:10.1016/j.pacs.2023.100510)
Supplement: Supplementary file 1 — Supplementary material [file mmc1.pdf]

# Azimuth mapping of fibrous tissue in linear dichroism-sensitive photoacoustic microscopy

Eunwoo Park [a], Yong-Jae Lee [b], Chulhong Kim [a], Tae Joong Eom [b, c, *]

[a] *Department of Convergence IT Engineering, Electrical Engineering, Mechanical Engineering, Medical Science and Engineering, Graduate School of Artificial Intelligence, and Medical Device Innovation Center, Pohang University of Science and Technology (POSTECH), Pohang, Gyeongbuk 37673, Republic of Korea*

[b] *Engineering Research Center for Color-Modulated Extra-Sensory Perception Technology, Pusan National University, Busan 46241, Republic of Korea*

[c] *Department of Congo-Mechatronics Engineering, Pusan National University, Busan 46241, Republic of Korea*

* Corresponding author: eomtj@pusan.ac.kr

## 1. Short-wave infrared photoacoustic microscopy (SWIR-PAM) imaging system performance

The SWIR-PAM system employed a light source with a central wavelength of 1540 nm for collagen-selective imaging. By setting a pulsed repetition rate (PRR) of 1 kHz, the imaging time was about 5 seconds per frame (with 4096-pixel A-lines and a 5-mm scanning range). In the acceleration region of unidirectional scanning, about 4% of the range was additionally scanned and dropped out to minimize image distortion. The imaging time varies depending on the field of view and the number of pixels and will be improved by using a high-PRR laser.

To identify the spatial resolutions of the imaging system, we used a 0.120-μm-thick chrome-masked glass resolution test target (R1DS1P, Thorlabs, USA). Fig. S1 shows the photoacoustic (PA) maximum amplitude projection image of the resolution test target. As shown in Fig. S1b and S1c, the axial and lateral resolutions were measured to be 115.00 μm and 3.35 μm, respectively. The axial resolution was estimated by obtaining the full width at half maximum (FWHM) of the Hilbert-transformed envelope of acquired A-line signals. A custom-made ring ultrasonic transducer has a -6 dB bandwidth of 8.2 MHz in silicone oil, resulting in a theoretical axial resolution of 107.32 μm. The speed of sound in silicone oil is about 1000 m/s. The speed of sound and the acoustic bandwidth vary depending on the acoustic matching medium, e.g. 1480 m/s and 13.5 Mhz in water. The lateral resolution was estimated by FWHM of the line spread function, the first derivative of the edge spread function.

As SWIR-PAM, collagen selectivity has been verified using tendon bundles extracted from a mouse tail (Fig. S1d). A three-dimensional (3D) PA image of label-free tendon bundles was obtained (Fig. S1e) and Fig. S1f shows the maximum amplitude projection image. The PA signals from the acoustic matching medium (water or silicone oil) were negligible.

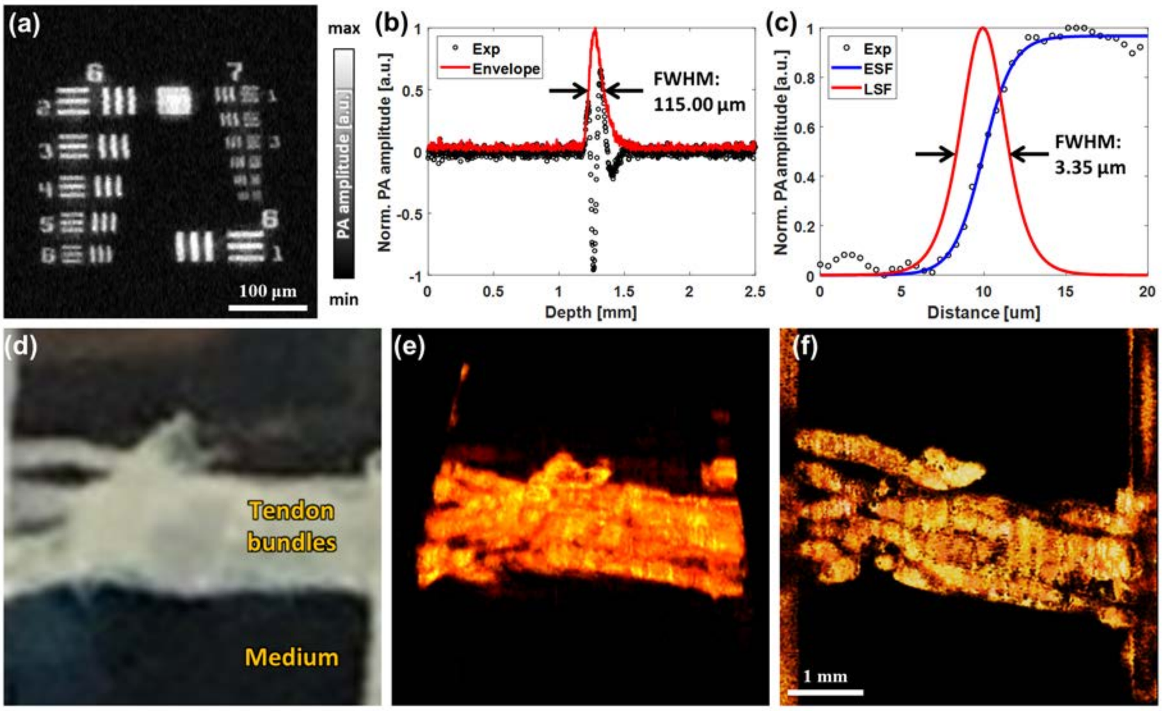


**Supplementary Fig. S1.** SWIR-PAM imaging system performance. (a) PA maximum amplitude projection image of the resolution test target. The measured (b) axial and (c) lateral resolutions of SWIR-PAM. (d) Photograph of tendon bundles, (e) Label-free 3D PA imaging, and (f) the projection view.

## 2. The feasibility of SWIR-DS-PAM

The SWIR-DS-PAM has been implemented to demonstrate vectorial optical absorption, which combines spectral contrast and polarization contrast. To confirm the feasibility of SWIR-DS-PAM, we conducted a material study by placing the linear polarizers (LP) and black polypropylene ($CH_3$-rich polymer) strap (PS) as high and low dichroic samples, respectively (Fig. S1a). Fig. S1b–d shows the SWIR-DS-PAM results of the phantom mixture. PS had a PA amplitude that was two orders of magnitude greater than LPs. However, the degree of dichroism was reversed, resulting in a 40-fold difference (e.g. LP~0.4 and PS<0.01). The azimuth of high-dichroic LP coincided with their own optical axes, whereas the low-dichroic PL exhibited a randomly distributed orientation.

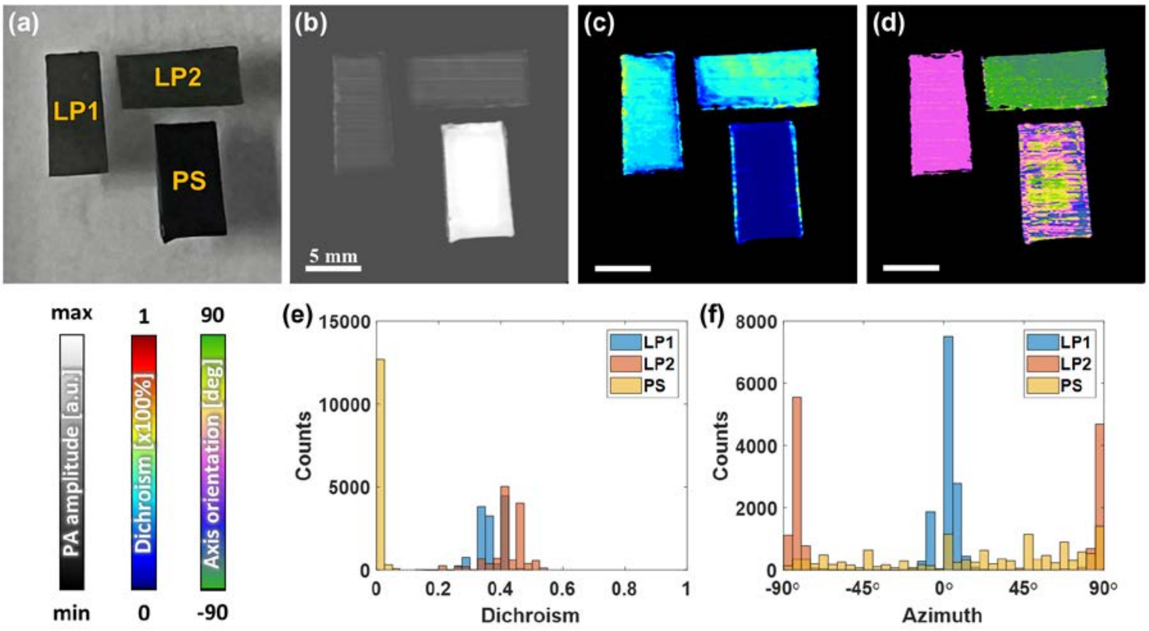


**Supplementary Fig. S2.** SWIR-DS-PAM imaging with mixed dichroic phantoms. (a) Photograph. LP, linear polarizer; PS, polypropylene strap. (b) Maximum amplitude projection image, (c) Dichroism map. (d) Azimuth map. (e–f) Histograms of phantoms' dichroism and azimuth, respectively.

## 3. DS-PA parameter-mapping validation using the trigonometric substitution

We demonstrated DS-PA parameter mapping according to the acquisition angle sets (Fig. S3). The azimuths were properly mapped as nylon wires were laid. The uniformity of dichroism was statistically analyzed at pixels corresponding to wires, and it was confirmed that the uniformity was improved at 4- and 6-angle sets.

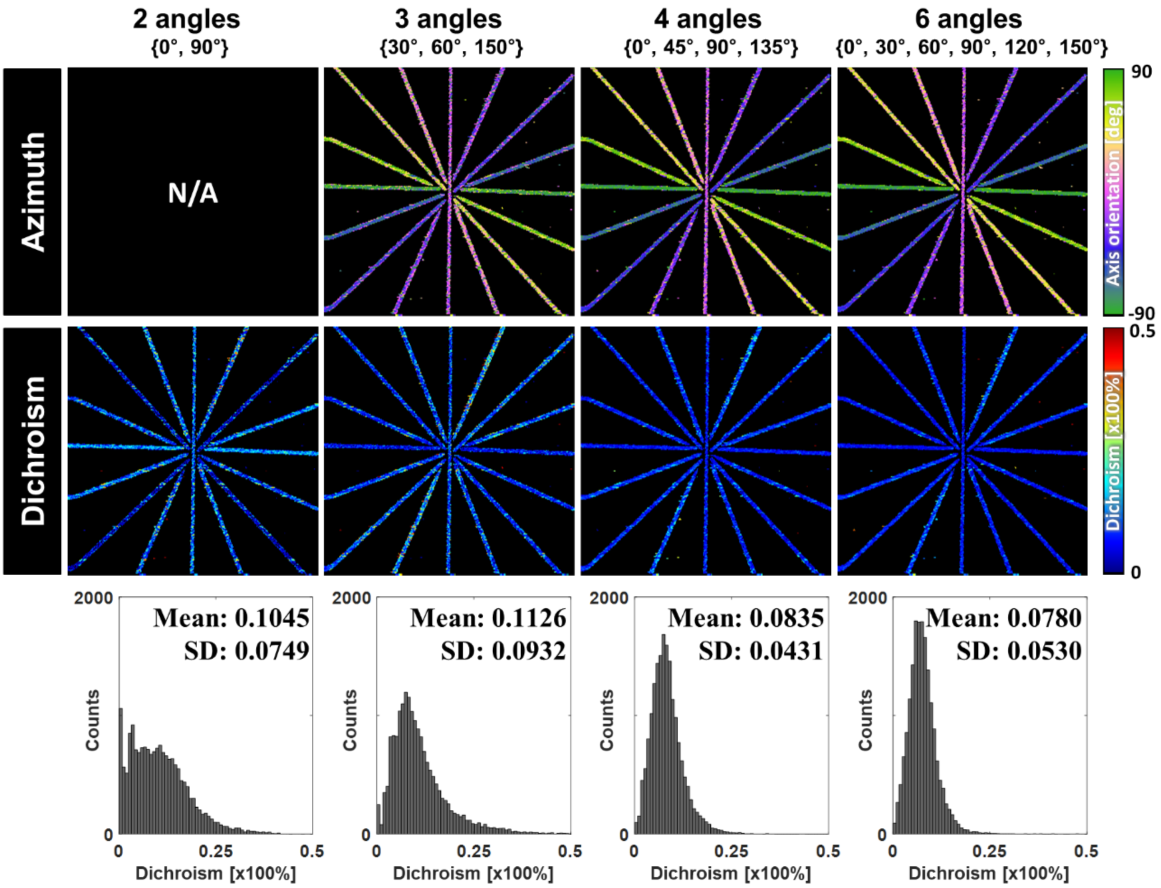


**Supplementary Fig. S3.** DS-PAM image reconstruction using the trigonometric method according to the number of applied angles and the histogram of dichroism distribution. Azimuth mapping with two angles is not available. Dichroism maps with an insufficient number of angles show low uniformity. SD, standard deviation.

## 4. Comparison of polarization-contrast optical imaging: DS-PAM and PM

To prove the feasibility of DS-PAM and mapping method, we compared the results with PM. According to Eq. (5) and Eq. (6), the dichroism and azimuth were matched to the DoLP and AoLP, respectively. Both polarization contrast (anisotropy) and spectral contrast (selectivity) were confirmed in DS-PAM.

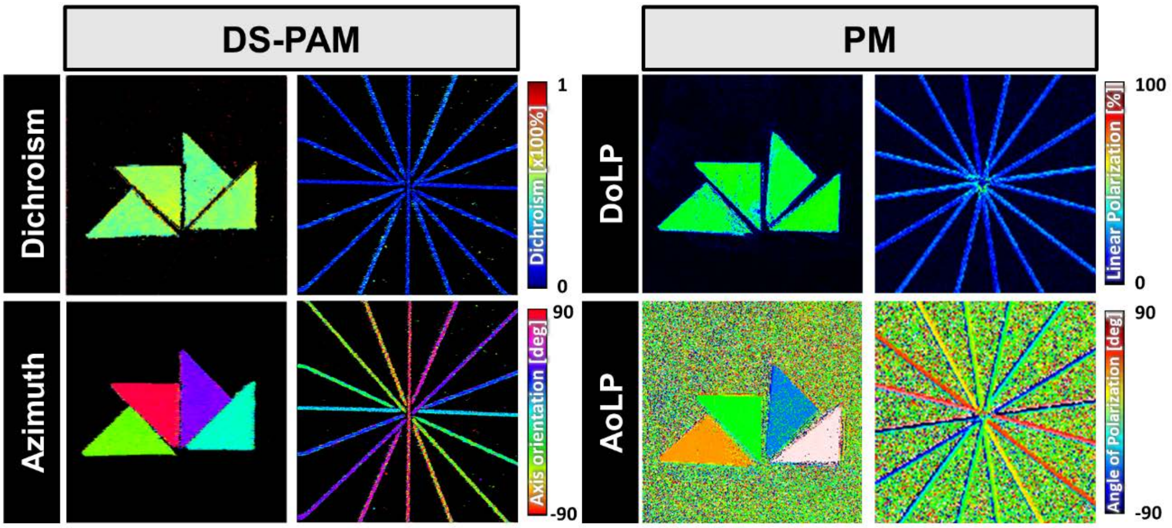


**Supplementary Fig. S4.** Polarization-contrast image comparison between DS-PAM and polarization microscopy (PM). DoLP, degree of linear polarization; AoLP, angle of linear polarization.
